# False-positive classification and associated factors in segmented macular layers and retinal nerve fiber layer analysis: Spectralis OCT deviation map study

**DOI:** 10.1038/s41598-023-33944-z

**Published:** 2023-04-25

**Authors:** Yun Jeong Lee, Ki Ho Park, Jin Wook Jeoung

**Affiliations:** Department of Ophthalmology, Seoul National University Hospital, Seoul National University College of Medicine, Seoul, Korea

**Keywords:** Glaucoma, Tomography

## Abstract

The rates, patterns and associated factors for false-positive classification of deviation maps by Cirrus optical coherence tomography (OCT) have been reported. However, research on OCT layer-by-layer deviation maps is lacking. We aimed to determine the rates and associated factors for false-positive classification of segmented macular layers and retinal nerve fiber layer (RNFL) deviation maps of Spectralis OCT, and to identify false-positive patterns on segmented macular layers deviation maps. 118 healthy eyes of 118 normal participants who had undergone Spectralis OCT imaging were included. False-positive classification was determined by the area and location of yellow or red color-coded regions on the deviation map. The false-positive rates on the deviation maps were the highest on the ganglion cell layer map, followed by the inner plexiform layer, retinal layer, and RNFL maps. More myopic/less hyperopic refractive error was a factor significantly associated with higher false-positive classification on the RNFL deviation map, and three false-positive patterns were found on the segmented macular layers deviation maps. Spectralis OCT deviation maps should be interpreted carefully to avoid misdiagnosis, especially for eyes with higher degrees of myopic refractive error on the RNFL map, for which purpose, recognizing the characteristic false-positive patterns would be helpful in clinical practice.

## Introduction

Optical coherence tomography (OCT) is a non-invasive, high-resolution imaging modality that allows for objective and quantitative structural assessment of the optic nerve head (ONH) and retina^1–3^, which utility makes it one of the most powerful tools for diagnosis and management of glaucoma^4–11^. For all its usefulness, however, OCT imaging often includes false-positive results of measurements. Considering the significant disease burden presented by glaucoma globally^12–15^, differentiating false-positives from true glaucomatous structural damage is vital in order to prevent unnecessary treatment and establish the most appropriate treatment plan for each patient.

A previous study has reported that the false-positive rates on ganglion cell analysis (GCA) and retinal nerve fiber layer (RNFL) maps by Cirrus OCT (Carl Zeiss Meditec, Dublin, CA, USA) were as high as about 40 and 30%, respectively, among healthy subjects, in which that of deviation map was the highest among GCA maps^16^. For detection of early glaucoma, the GCA deviation map also has shown the highest false-positive rate among GCA maps by Cirrus OCT, though also the highest sensitivity^17^, which fact strongly suggests the need for careful interpretation of results to avoid misdiagnosis.

Recently, the Spectralis OCT (Heidelberg Engineering, Heidelberg, Germany) Glaucoma Module Premium Edition (GMPE) software newly added thickness deviation maps for segmented retinal layers including the ganglion cell layer (GCL), inner plexiform layer (IPL), retinal nerve fiber layer (RNFL) and the full retina, which reveal regions and patterns having significantly different thickness measurement values from those in the built-in internal normative database.

Although there has been research on the rates, patterns and associated factors for false-positive classification of deviation maps by Cirrus OCT^16–19^, studies on OCT layer-by-layer deviation maps are lacking. The aim of our study, therefore, was to determine the rates and associated factors for false-positive classification of segmented macular layers and RNFL deviation maps by Spectralis OCT and to identify false-positive patterns on segmented macular layers deviation maps.

## Results

A total of 118 healthy eyes of 118 normal participants were included in our study. The demographics and characteristics of the participants are shown in Table 1. The mean age, axial length (AXL), and refractive error were 55.8 ± 15.0 years, 24.7 ± 1.7 mm, and − 2.4 ± 3.5 diopters, respectively. The mean Fovea-to-Bruch’s membrane opening-Center (FoBMOC) axis and Bruch’s membrane opening (BMO) area were − 6.4° ± 3.5° and 2.39 ± 0.57 mm^2^, respectively. Also, the average Bruch’s membrane opening-minimum rim width (BMO-MRW) was 252.7 ± 41.4 µm, and the average RNFL thickness was 23.8 ± 2.6 µm.

**Table 1:** Demographics and characteristics of participants.

	Normal (n = 118)
Age (year)	55.8 ± 15.0
Male [n (%)]	59 (50.0)
Right eyes [n (%)]	70 (59.3)
IOP (mmHg)	13.7 ± 2.5
CCT (µm)	548.7 ± 31.3
AXL (mm)	24.7 ± 1.7
Refractive error (D)	− 2.4 ± 3.5
FoBMOC axis (°)	− 6.4 ± 3.5
BMO area (mm^2^)	2.39 ± 0.57
BMO-MRW (µm)	252.7 ± 41.4
RNFL thickness (µm)	23.8 ± 2.6
VF MD (dB)	− 0.33 ± 1.30
VF PSD (dB)	1.64 ± 0.67

*AXL* axial length, *BMO* Bruch’s membrane opening, *CCT* central corneal thickness, *D* diopter, *FoBMOC* Fovea-to-BMO-Center, *IOP* intraocular pressure, *MD* mean deviation, *MRW* minimum rim width, *PSD* pattern standard deviation, *RNFL* retinal nerve fiber layer, *VF* visual field.

Data are mean ± standard deviation unless otherwise indicated.

### False-positive rates on segmented macular layers and retinal nerve fiber layer analysis maps

**Figure 1:**
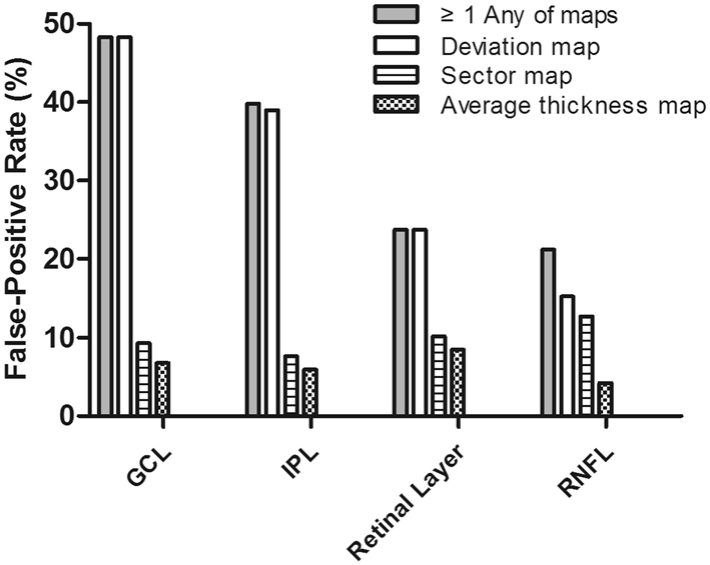
False-positive rates on segmented macular layers (ganglion cell layer [GCL], inner plexiform layer [IPL] and retinal layer) and retinal nerve fiber layer (RNFL) analysis maps of Spectralis OCT.

In the layer-by-layer analysis (Fig. 1), for the deviation map, the false-positive rate was the highest for GCL (57 eyes (48.3%)), followed by IPL (46 eyes (39.0%)), retinal layer (28 eyes (23.7%)), and RNFL (18 eyes (15.3%)). The same trend was found for the overall false-positive rate (≥ 1 abnormal color codes on any of the 3 maps): the GCL map was the highest (57 eyes (48.3%)), followed by the IPL (47 eyes (39.8%)), retinal layer (28 eyes (23.7%)), and RNFL (25 eyes (21.2%)) maps. As for the sector map, however, RNFL showed the highest false-positive rate (15 eyes (12.7%)), followed by retinal layer (12 eyes (10.2%)), GCL (11 eyes (9.3%)) and IPL (9 eyes (7.6%)). For the average thickness map, the retinal layer had the highest false-positive rate (10 eyes (8.5%)), followed by GCL (8 eyes (6.8%)), IPL (7 eyes (5.9%)), and RNFL (5 eyes (4.2%)).

**Figure 2:**
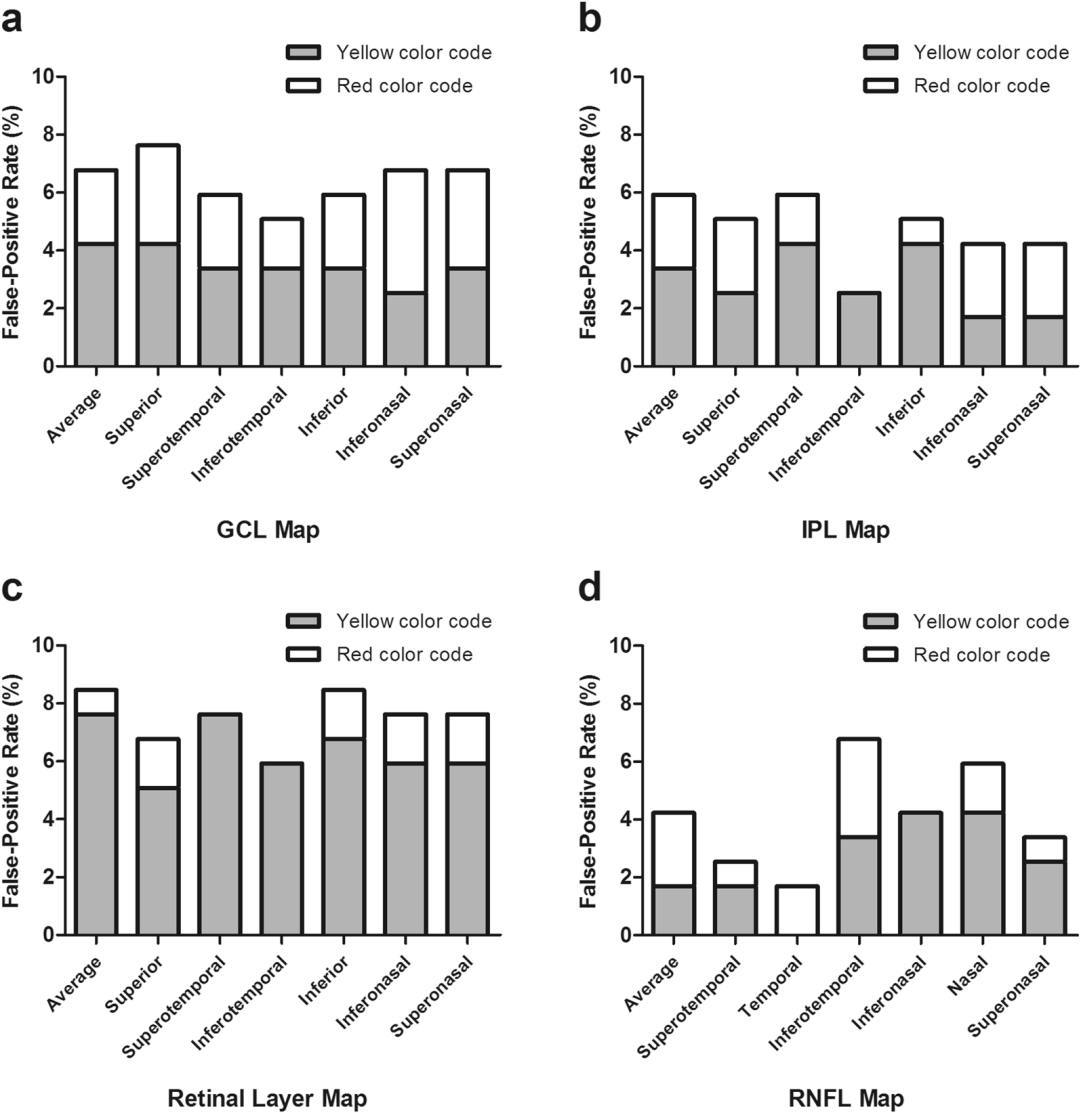
False-positive rates on sector maps of Spectralis OCT. The false-positive rates are represented in yellow (outside the 95% normal limit) and red (outside the 99% normal limit) color codes on the ganglion cell layer (GCL) map (**a**), inner plexiform layer (IPL) map (**b**), retinal layer map (**c**) and retinal nerve fiber layer (RNFL) map (**d**).

Among the 3 analysis maps (deviation, sector, and average thickness), the deviation map showed the highest false-positive rate, followed by the sector map and the average thickness map, in all of the segmented macular layers (GCL, IPL, and retinal layer) and RNFL (Fig. 1). On the sector map, for GCL, the superior quadrant had the highest false-positive rate (9 eyes (7.6%)), followed by the inferonasal and superonasal (8 eyes (6.8%) each), superotemporal and inferior (7 eyes (5.9%) each), and inferotemporal (6 eyes (5.1%)) quadrants (Fig. 2a); for IPL, the superotemporal quadrant had the highest false-positive rate (7 eyes (5.9%)), followed by the superior and inferior (6 eyes (5.1%) each), inferonasal and superonasal (5 eyes (4.2%) each), and inferotemporal (3 eyes (2.5%)) quadrants (Fig. 2b); for the retinal layer, the inferior quadrant had the highest false-positive rate (10 eyes (8.5%)), followed by the superotemporal, inferonasal and superonasal (9 eyes (7.6%) each), superior (8 eyes (6.8%)), and inferotemporal (7 eyes (5.9%)) quadrants (Fig. 2c); for the RNFL, the inferotemporal quadrant had the highest false-positive rate (8 eyes (6.8%)), followed by the nasal (7 eyes (5.9%)), inferonasal (5 eyes (4.2%)), superonasal (4 eyes (3.4%)), superotemporal (3 eyes (2.5%)), and temporal (2 eyes (1.7%)) quadrants (Fig. 2d).

### False-positive patterns on segmented macular layers deviation maps

**Figure 3:**
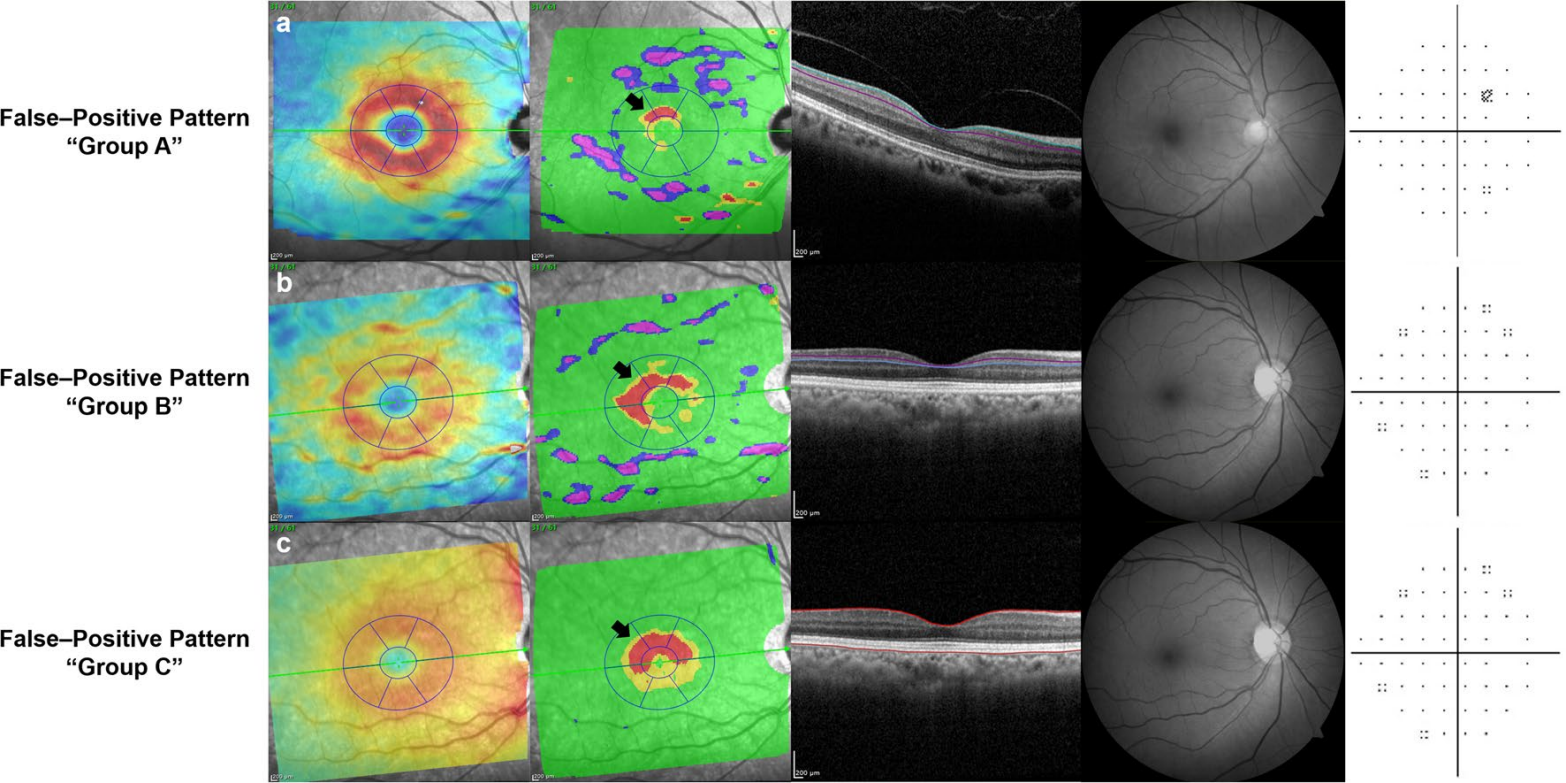
False-positive patterns on deviation maps of segmented macular layers on Spectralis OCT. Each labeled row (**a**–**c**), from left to right, consists of a thickness map, a deviation map, a corresponding B-scan with colored lines demarcating the segmented layer, red-free retinal nerve fiber layer (RNFL) photography and Humphrey (Carl Zeiss Meditec, Dublin, CA, USA) C24–2 visual field. (**a**) Healthy eye of 58-year-old female with pattern group A (island shape circumpassing less than 180° in area) on ganglion cell layer (GCL) deviation map. (**b**) Healthy eye of 66-year-old female with pattern group B (hook shape circumpassing more than 180° but less than 360° in area) on inner plexiform layer (IPL) deviation map. (**c**) Healthy eye of 66-year-old female with pattern group C (donut shape around inner annulus, circumpassing 360° in area) on retinal layer deviation map.

**Figure 4:**
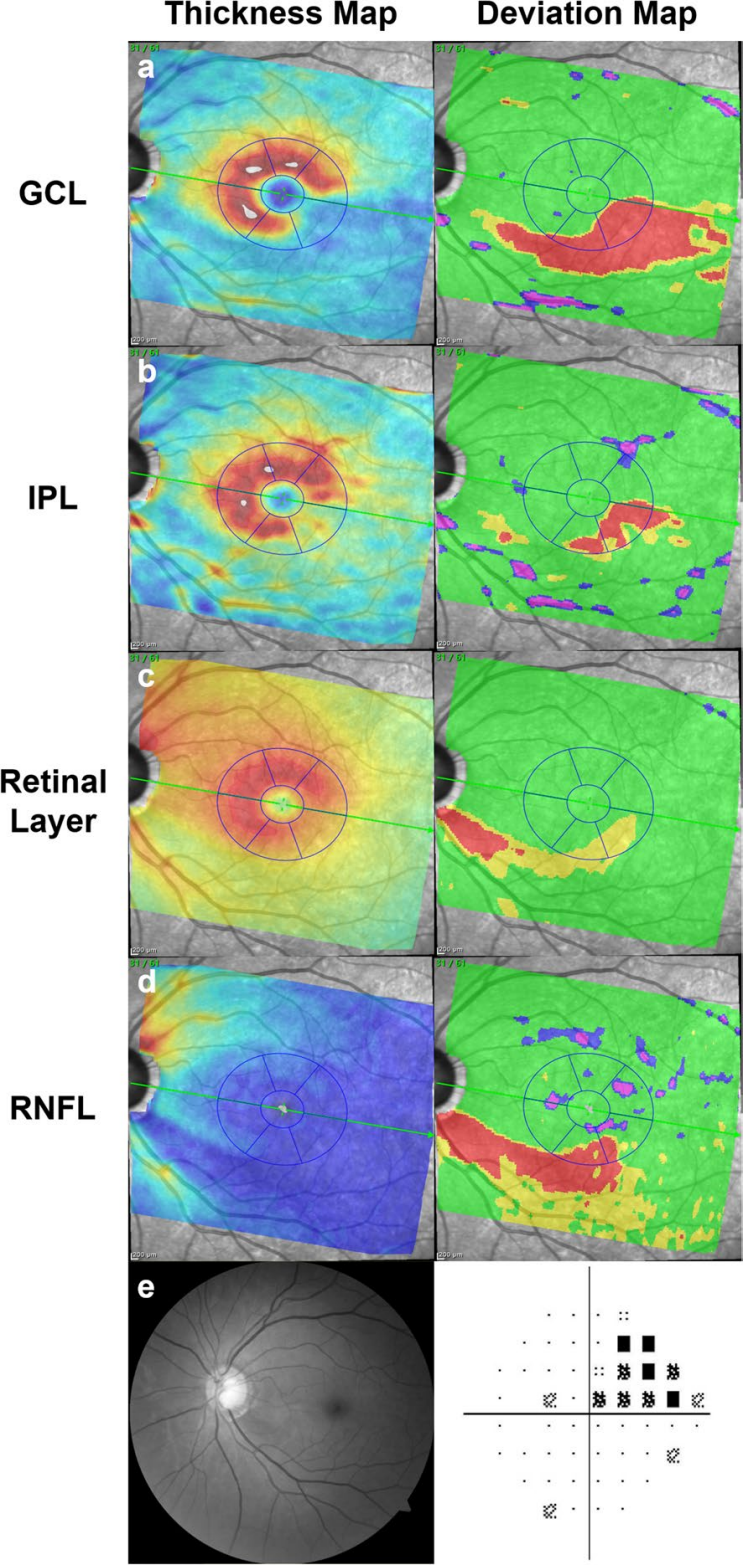
Spectralis OCT deviation maps of segmented macular layers and retinal nerve fiber layer (RNFL) in glaucomatous eye. Each labeled row (**a**–**d**), from left to right, consists of a thickness map and a deviation map, except for **e** with red-free RNFL photography and Humphrey (Carl Zeiss Meditec) C24–2 visual field. A patient with open-angle glaucoma showing inferior localized glaucomatous defects on the ganglion cell layer (GCL) (**a**), inner plexiform layer (IPL) (**b**), retinal layer (**c**), RNFL maps (**d**) and red-free RNFL photography, with corresponding superior visual field defect (**e**).

The patterns of false-positives on the segmented macular layers deviation maps were classified into 3 groups according to the shape and area of the abnormal color-coded area: group A (island shape circumpassing less than 180° in area) (Fig. 3a); group B (hook shape circumpassing more than 180° but less than 360° in area) (Fig. 3b); group C (donut shape around inner annulus, circumpassing 360° in area) (Fig. 3c). As for the determination of the false-positive-pattern groups, we found almost perfect interobserver agreement (kappa = 0.878 for GCL, 0.855 for IPL, and 0.891 for retinal layer, all *p*s < 0.001). Figure 4 reveals the true glaucomatous structural damage on the segmented macular layers and RNFL deviation maps.

### Comparison of demographics and characteristics among different false-positive-pattern groups on segmented macular layers deviation maps

Demographics and characteristics were analyzed and compared among the groups for the respective segmented macular layers deviation maps, as shown in Supplementary Tables 1–3. For all of the layers, group A was the most common (43 (75.4%), 39 (84.8%), and 12 (42.9%) eyes for GCL, IPL, and retinal layer, respectively), followed by group B (9 (15.8%), 5 (10.9%), and 9 (32.1%) eyes for GCL, IPL, and retinal layer, respectively) and group C (5 (8.8%), 2 (4.3%), and 7 (25.0%) eyes for GCL, IPL, and retinal layer, respectively). Among the characteristics analyzed, age was the only factor that showed significant differences among groups on the GCL deviation map (*p* = 0.001), group C being the oldest, followed by groups B and A (Supplementary Table 1). An intergroup comparison by post hoc test revealed that there were significant age differences between groups A and B (*p* = 0.016) and groups A and C (*p* < 0.001). On the IPL deviation map, age and axial length showed significant differences among the groups (*p* = 0.013 for age and *p* = 0.024 for axial length) (Supplementary Table 2). As was the case with the GCL deviation map, group C was the oldest, followed by groups B and A, the contrast between groups A and B being significant (*p* = 0.006). Axial length was the longest in group C, followed by groups A and B, that in group A being significantly longer than that in group B (*p* = 0.001). In summary, the IPL deviation map indicated that the patients of group B were significantly older and had notably shorter axial length compared with those of group A. On the retinal layer deviation map, no remarkable differences in factors were found among the groups (Supplementary Table 3).

### Factors associated with false-positive classification on segmented macular layers and retinal nerve fiber layer analysis maps

Table 2 presents the factors associated with false-positives on the segmented macular layers and RNFL analysis maps. In the univariate analysis, younger age (odds ratio [OR] 0.96; 95% confidence interval [CI] 0.93–0.99; *p* = 0.006), longer axial length (OR 1.42; 95% CI 1.05–1.91; *p* = 0.021), more myopic/less hyperopic refractive error (OR 0.82; 95% CI 0.71–0.94; *p* = 0.004) and larger BMO area (OR 2.47; 95% CI 1.16–5.28; *p* = 0.019) were associated with higher rate of false-positives on ≥ 1 of any of the RNFL maps. On the deviation map, younger age (OR 0.96; 95% CI 0.93–0.99; *p* = 0.022), longer axial length (OR 1.56; 95% CI 1.12–2.17; *p* = 0.009) and more myopic/less hyperopic refractive error (OR 0.83; 95% CI 0.71–0.97; *p* = 0.017) were associated with higher rate of false-positives for RNFL. On the sector map, younger age (OR 0.95; 95% CI 0.92–0.98; *p* = 0.004), more myopic/less hyperopic refractive error (OR 0.78; 95% CI 0.65–0.92; *p* = 0.004) and larger BMO area (OR 3.00; 95% CI 1.26–7.10; *p* = 0.013) were significant factors for RNFL. On the average thickness map, older age (OR 1.12; 95% CI 1.02–1.23; *p* = 0.022) was a significant factor for IPL, as was larger BMO area (OR 7.31; 95% CI 1.93–27.63; *p* = 0.003) for RNFL.

**Table 2:** Factors associated with false-positive classification on segmented macular layers and retinal nerve fiber layer analysis maps in univariate and multivariate logistic regression analyses.

	≥ 1 of any of maps				Deviation map				Sector map				Average thickness map			
	GCL	IPL	Retinal layer	RNFL	GCL	IPL	Retinal layer	RNFL	GCL	IPL	Retinal layer	RNFL	GCL	IPL	Retinal layer	RNFL
Univariate analysis																
Age	1.00 (0.786)	1.00 (0.758)	1.02 (0.286)	**0.96 (0.006)**	1.00 (0.786)	1.00 (0.719)	1.02 (0.286)	**0.96 (0.022)**	1.04 (0.105)	1.05 (0.096)	1.01 (0.481)	**0.95 (0.004)**	1.03 (0.308)	**1.12 (0.022)**	1.00 (0.861)	0.98 (0.472)
Gender (male)	0.62 (0.198)	1.43 (0.348)	1.46 (0.388)	0.90 (0.822)	0.62 (0.198)	1.33 (0.451)	1.46 (0.388)	1.00 (1.000)	1.22 (0.752)	2.11 (0.307)	1.45 (0.544)	0.86 (0.782)	1.73 (0.468)	1.36 (0.698)	1.00 (1.000)	1.53 (0.650)
Laterality (right)	0.97 (0.944)	1.14 (0.736)	1.36 (0.479)	1.19 (0.703)	0.97 (0.944)	1.04 (0.912)	1.36 (0.479)	1.20 (0.724)	1.24 (0.735)	1.92 (0.351)	1.05 (0.941)	1.80 (0.290)	1.50 (0.580)	1.10 (0.904)	1.51 (0.533)	2.27 (0.380)
IOP	1.14 (0.094)	0.98 (0.756)	1.09 (0.287)	0.93 (0.424)	1.14 (0.094)	0.98 (0.737)	1.09 (0.287)	0.91 (0.407)	1.09 (0.430)	1.05 (0.704)	1.10 (0.361)	1.01 (0.940)	1.01 (0.942)	1.11 (0.434)	1.08 (0.511)	0.95 (0.799)
CCT	1.00 (0.545)	1.00 (0.572)	0.99 (0.187)	1.00 (0.555)	1.00 (0.545)	1.00 (0.803)	0.99 (0.187)	0.99 (0.361)	1.00 (0.753)	1.00 (0.928)	1.00 (0.720)	1.00 (0.570)	1.01 (0.468)	1.00 (0.929)	1.00 (0.827)	0.98 (0.164)
AXL	1.08 (0.495)	1.10 (0.435)	1.15 (0.320)	**1.42 (0.021)**	1.08 (0.495)	1.08 (0.547)	1.15 (0.320)	**1.56 (0.009)**	0.72 (0.187)	0.70 (0.251)	0.98 (0.922)	1.34 (0.112)	0.87 (0.622)	0.55 (0.129)	1.24 (0.352)	1.25 (0.456)
Refractive error	0.98 (0.717)	0.98 (0.720)	0.90 (0.129)	**0.82 (0.004)**	0.98 (0.717)	0.98 (0.720)	0.90 (0.129)	**0.83 (0.017)**	1.19 (0.199)	1.21 (0.274)	0.94 (0.537)	**0.78 (0.004)**	1.07 (0.627)	1.48 (0.135)	0.87 (0.195)	0.78 (0.110)
FoBMOC axis	1.07 (0.200)	1.01 (0.863)	1.08 (0.236)	0.93 (0.287)	1.07 (0.200)	1.02 (0.699)	1.08 (0.236)	0.94 (0.392)	1.04 (0.639)	0.98 (0.816)	1.10 (0.268)	1.01 (0.891)	1.02 (0.859)	0.99 (0.950)	1.13 (0.197)	0.88 (0.342)
BMO area	0.64 (0.189)	0.80 (0.519)	0.65 (0.307)	**2.47 (0.019)**	0.64 (0.189)	0.73 (0.365)	0.65 (0.307)	1.21 (0.658)	0.43 (0.234)	0.81 (0.752)	0.42 (0.201)	**3.00 (0.013)**	0.46 (0.329)	1.17 (0.815)	0.38 (0.195)	**7.31 (0.003)**
Multivariate analysis																
Age	–	–	–	0.98 (0.263)	–	–	–	0.99 (0.593)	0.98 (0.590)	–	–	0.92 (0.190)	–	1.07 (0.570)	–	–
Gender (male)	0.70 (0.363)	–	–	–	0.70 (0.363)	–	–	–	–	–	–	–	–	–	–	–
IOP	1.15 (0.082)	–	–	–	1.15 (0.082)	–	–	–	–	–	–	–	–	–	–	–
CCT	–	–	0.99 (0.130)	–	–	–	0.99 (0.130)	–	–	–	–	–	–	–	–	0.99 (0.812)
AXL	–	–	–	0.82 (0.560)	–	–	–	1.13 (0.740)	0.63 (0.108)	–	–	0.23 (0.171)	–	0.19 (0.053)	–	–
Refractive error	–	–	0.89 (0.100)	**0.83 (0.017)**	–	–	0.89 (0.100)	**0.82 (0.016)**	0.97 (0.919)	–	–	**0.47 (0.019)**	–	0.84 (0.762)	0.84 (0.138)	0.89 (0.507)
FoBMOC axis	–	–	–	–	–	–	–	–	–	–	–	–	–	–	1.25 (0.068)	–
BMO area	0.64 (0.201)	–	–	1.71 (0.250)	0.64 (0.201)	–	–	–	–	–	–	1.46 (0.489)	–	–	0.08 (0.065)	4.36 (0.075)

*AXL* axial length, *BMO* Bruch’s membrane opening, *CCT* central corneal thickness, *FoBMOC* Fovea-to-BMO-Center, *GCL* ganglion cell layer, *IOP* intraocular pressure, *IPL* inner plexiform layer, *RNFL* retinal nerve fiber layer.

Data are odds ratio with *p* value in parenthesis.

Significant values are in bold.

In the multivariate analysis, more myopic/less hyperopic refractive error was the only factor significantly associated with higher false-positive classification on ≥ 1 of any of the maps or the deviation or sector maps of RNFL (OR 0.83; 95% CI 0.72–0.97; *p* = 0.017 on ≥ 1 of any of the maps; OR 0.82; 95% CI 0.70–0.96; *p* = 0.016 on deviation map; OR 0.47; 95% CI 0.25–0.88; *p* = 0.019 on sector map).

## Discussion

In the present study, we investigated the rates and associated factors for false-positive classification of segmented macular layers and RNFL deviation maps by Spectralis OCT and identified the false-positive patterns on segmented macular layers deviation maps. Our results revealed that the false-positive rate on the deviation map was the highest for GCL (57 eyes (48.3%)), followed by IPL (46 eyes (39.0%)), retinal layer (28 eyes (23.7%)), and RNFL (18 eyes (15.3%)), and was also the highest among the 3 analysis maps for all of the segmented macular layers and RNFL maps. In addition, more myopic/less hyperopic refractive error was an associated factor for false-positive classification on the deviation map of RNFL, and 3 characteristic false-positive patterns of island, hook and donut shape were found on the segmented macular layers deviation maps.

These results are in line with a previous study by Kim et al.^16^, which reported a higher rate of false-positives on a deviation map for GCA (37.5%) than for RNFL (14.4%) by Cirrus OCT. Although the false-positive rate on the RNFL deviation map in our study (15.3%) is similar to that reported by Kim et al.^16^ (14.4%) or Kim et al.^18^ (20.1%), that for segmented macular layers (GCL map (48.3%) or IPL map (39.0%)) is higher than that of Hwang et al.^17^ (28.0%) or Kim et al.^16^ (37.5%) with GCA maps on Cirrus OCT. The factors that contributed to the differences might be the cancellation effect derived from analyzing both GCL and IPL layers together on the GCA map, which could have resulted in fewer false-positive results than when analyzing the layers separately. As for the comparison of false-positive rates among the analysis maps, our results are in accordance with the literature, wherein the deviation map showed the highest false-positive rates among GCA maps on Cirrus OCT, followed by the sector map and the average thickness map^16,17^.

The discrepancy between the analysis maps could be explained by differences in the analysis methods, where the deviation map reveals abnormal findings as they are, whereas the sector map and, moreover, the average thickness map present layer thickness on average, which does not reflect the true distribution of layer fibers. Regarding the RNFL analysis maps, the same trend as in earlier studies with Cirrus OCT^16,17^ was found: the false-positive rates on the deviation map were higher than those on the quadrant map. A previous study using Spectralis OCT RNFL analysis maps reported false-positive rates of 10% on the RNFL sector map^19^, which is similar to our finding of 12.7%. However, because they did not include a deviation map in their analyses, comparisons among the various analysis maps are difficult.

The present false-positive patterns are similar to those found by Kim et al. with Cirrus OCT^16^, which also revealed the round donut and isolated island-like shapes. When comparing the patient characteristics among the pattern groups, groups B and C were significantly older than group A on the GCL deviation map, while group B was significantly older and had notably shorter axial length than did group A on the IPL deviation map. These findings indicate that both characteristic false-positive patterns and factors showing differences among groups should be considered when interpreting the deviation map of segmented macular layers.

In our study, more myopic/less hyperopic refractive error was the only factor significantly associated with higher false-positive classification on ≥ 1 of any of the maps or the deviation or sector maps of RNFL. The results are in accordance with earlier studies demonstrating that myopic eyes are prone to false-positive results on RNFL analysis maps by establishing an association of longer axial length with false-positive classification^16,18^. Regarding the present segmented macular layers classification, however, no significant factors were found to be associated with false-positive classification. In light of previous studies that determined longer axial length, higher degree of myopic refractive error and larger fovea-disc angle to be factors associated with false-positive GCA findings on Cirrus OCT^16,17^, our findings might have arisen from differences in the degree and distribution of myopia and fovea-disc angle among our study population.

Analysis of segmented macular layers using Spectralis OCT is meaningful since the macular GCL, consisting of retinal ganglion cell bodies, has been reported to have comparable diagnostic ability to peripapillary RNFL for early glaucoma^20^, and the IPL, which contains retinal ganglion dendrites, has been found to be associated with glaucoma degree^21^. Indeed, these findings suggest that segmented macular layer parameters could serve as potential biomarkers for evaluation of glaucoma. In this regard, determination of false-positives in segmented macular layers should come into its own in the future when layer-by-layer analysis has become more generalized for assessment of glaucoma in clinical practice.

The strength of our study is that it is, to our best knowledge at least, the first to have determined the false-positive rates, patterns, and associated factors for segmented macular layers. Another main strength is that we compared false-positives among the various analysis maps of Spectralis OCT. The limitations of this study include a lack of comparison for false-positives among groups of different clinical characteristics such as SE or AXL. Further studies of prospective design that conduct intergroup comparisons will further expand our knowledge and understanding of false-positive classification on Spectralis OCT. Also, analyses on the distribution of false-positives as a function of the abnormality area threshold would be meaningful, considering different robustness of each layer. Future analyses to computationally identify the false-positive-pattern groups are required as well, given that they were obtained by visual observation. Moreover, because there have been only a few studies evaluating false-positives on OCT, each employing different definitions of false-positive and different types of analysis map (thus rendering inter-study comparison problematic), criteria standardization for both false-positives and analysis maps in OCT is required for future research. Additionally, further refinement of Spectralis OCT’s analysis software to enable direct provision of area and length measurements on deviation maps (rather than continuing to rely on imported software) will increase and broaden its utilization in clinical practice. Finally, the false-positive results might have arisen from the non-representative normative data of the OCT device (adopted from a European population), the current study’s participants having been all Asian. Future development of Spectralis OCT software that provides a normative database reflecting patients’ racial/ethnic differences is required.

In conclusion, when interpreting Spectralis OCT deviation maps, care should be taken to avoid misdiagnosis, especially for eyes with higher degrees of myopic refractive error on the RNFL map, for which purpose, recognizing the characteristic false-positive patterns definitely would be helpful in clinical practice.

## Methods

This retrospective, cross-sectional study enrolled subjects who had visited Seoul National University Hospital (SNUH) in South Korea from October 2020 to September 2021. The study was approved by the Institutional Review Board of SNUH (IRB No. 2111-139-1275), and the study protocol followed the tenets of the Declaration of Helsinki. Informed consent was waived by the IRB of SNUH.

### Participants

All of the participants underwent a complete ophthalmologic examination during their routine health checkup, including measurements of best-corrected visual acuity (BCVA), refraction, central corneal thickness (Pocket II Pachymeter Echograph; Quantel Medical, Clermont-Ferrand, France) and AXL (Axis II PR; Quantel Medical, Bozeman, MT, USA), as well as Goldmann applanation tonometry, slit-lamp biomicroscopy, gonioscopy, dilated fundus examination, stereo disc photography, red-free RNFL photography (Visucam; Carl Zeiss Meditec), spectral-domain OCT (SD-OCT; Spectralis OCT, Heidelberg Engineering) and standard automated perimetry (Humphrey Field Analyzer II; 24–2 Swedish Interactive Threshold Algorithm; Carl Zeiss Meditec).

For inclusion in the present study, subjects were required to meet the following criteria: BCVA of 20/40 or better, as well as normal anterior chamber and open angle on slit-lamp and gonioscopic examinations, respectively. The eligibility criteria for healthy eyes were as follows: intraocular pressure (IOP) of 21 mmHg or less, no history of increased IOP, optic disc of normal appearance (i.e., cup-to-disc ratio ≤ 0.5, cup-to-disc ratio asymmetry ≤ 0.2 between the eyes, no neuroretinal rim notching or thinning, optic disc hemorrhage, or localized pallor), no RNFL defect on red-free RNFL photography, and normal visual field (VF) results. Color disc and red-free RNFL photographs were evaluated by 2 glaucoma specialists (Y.J.L. and J.W.J.) masked to all clinical information.

Participants with a history of ocular surgery other than uncomplicated cataract surgery, ocular inflammation or trauma, retinal diseases (e.g., diabetic retinopathy, retinal vein occlusion, or age-related macular degeneration) or neurologic diseases (e.g., pituitary tumor) that could cause VF defect were excluded.

### Spectralis OCT imaging

All of the subjects underwent Spectralis OCT imaging with GMPE software, which incorporates an Anatomic Positioning System (APS) that automatically identifies two fixed anatomical landmarks—the center of the BMO and the fovea, creating a FoBMOC axis—and aligns scans relative to the patient’s individual FoBMOC axis, hence improving the accuracy of measurement.

Analysis of the ONH (24 high-resolution 15° radial scans, each averaged from 27 B-scans) automatically defined the BMO and internal limiting membrane (ILM), and the neuroretinal rim was assessed according to the BMO-MRW, which was calculated from the BMO to the nearest point on the ILM. ONH parameters including BMO area and BMO-MRW were analyzed.

Circular scans of 3.5 mm diameter were performed using circumpapillary RNFL GMPE to obtain RNFL thicknesses around the disc, providing their average and sectoral (superotemporal, superonasal, nasal, inferonasal, inferotemporal, and temporal) measurement values. Color codes of green (within normal range), yellow (outside 95% normal limit), or red (outside 99% normal limit) were assigned based on comparison with the internal normative database.

Macular imaging was performed with posterior pole scans consisting of 61 B-scans comprising 768 A-scans each captured on a 30° × 25° square centered on the macula, tilted parallel to the FoBMOC axis. Images were automatically segmented for the GCL, IPL, RNFL, and the full retinal layer using built-in software provided by the device manufacturer (Heidelberg Engineering). After segmentation, thickness maps were computed for each layer, and the average and sectoral (superotemporal, superior, superonasal, inferonasal, inferior, and inferotemporal) thickness values were provided. The same green, yellow and red color codes as used for the circumpapillary RNFL (see above) were assigned, again based on comparison with the internal normative database. The normative database adopted for the sector map was developed based on each sector as defined by a macula grid (6-sector Garway-Heath grid) on OCT scans. Also, deviation maps were provided for each layer, revealing regions and patterns having significantly thinner or thicker measurement values relative to those in the reference database by color-highlighting the areas outside the normal limits: red and yellow representing percentile values of < 1% and < 5%, respectively. Only good-quality OCT images, defined as those with quality score ≥ 15, clearly visible and delineated macular and circumpapillary RNFL layers, without misalignment, motion or blink artifacts, defocus, or segmentation error (as checked manually), were included.

### False-positive classification of segmented macular layers and retinal nerve fiber layer on analysis maps

Two glaucoma specialists (Y.J.L. and J.W.J.) masked to all clinical information identified the false-positive color codes on segmented macular layers (GCL, IPL, and retinal layer) and RNFL maps as well as the patterns of false-positives on segmented macular layers deviation maps. The yellow- or red-coded average thickness map and a sector map with ≥ 1 yellow- or red-colored sectors were considered abnormal. To measure the area and location of the abnormal color-coded regions on the deviation map, we imported OCT images into a customized software (ImageJ; National Institutes of Health, Bethesda, MD, USA). Using the same criteria as in our former study reporting false-positives on Cirrus OCT^16^, an abnormal GCL, IPL, or retinal layer deviation map was arbitrarily defined as a contiguous yellow- or red color-coded region of at least 0.144 mm^2^ in area (equivalent to 10 superpixels in area on Cirrus OCT) with a boundary more than 0.12 mm (equivalent to 1 superpixel on Cirrus OCT) from the inner annulus. To eliminate artifacts, images in which the area of the largest color-coded region was less than 0.144 mm^2^ were excluded. An abnormal RNFL deviation map was defined as a wedge-shaped, radiating yellow- or red-colored pattern of ≥ 1.2 mm (equivalent to 10 superpixels on Cirrus OCT) in length and ≥ 0.24 mm (equivalent to 2 superpixels on Cirrus OCT) in width.

### Statistical analyses

The interobserver agreement for determination of the false-positive-pattern groups was assessed using kappa statistics. Descriptive statistics were done to represent the demographic and patient-characteristic data. The rate of false-positive results was calculated as the number of eyes with abnormal segmented macular layers or RNFL maps divided by the total number of eyes. An overall false-positive rate was determined by the number of eyes with ≥ 1 segmented macular layers or RNFL maps (deviation, sector, or average thickness) with abnormal color codes. Fisher's exact test was used to compare the categorical variables among the subgroups with different false-positive patterns on segmented macular layer deviation maps, and the Kruskal–Wallis test was utilized for continuous variables along with the Mann–Whitney U post hoc test for their pairwise comparison. Univariate and multivariate logistic regression analyses were performed to determine the factors associated with false-positive classification on analysis maps. Only variables with *p* < 0.20 as determined in the univariate logistic analyses were further analyzed in multivariate analyses. All statistical analyses were performed using the Statistical Package for the Social Sciences version 25.0 (IBM Corp., Armonk, NY, USA). In the analysis, *p* values < 0.05 were considered statistically significant.

## Supplementary Information

Supplementary Information.

## Data Availability

The data that support the findings of this study are available from the corresponding author upon reasonable request.

## Abbreviations

APS: Anatomic Positioning System
AXL: Axial length
BCVA: Best-corrected visual acuity
BMO: Bruch’s membrane opening
BMO-MRW: Bruch’s membrane opening-minimum rim width
CI: Confidence interval
FoBMOC: Fovea-to-Bruch’s membrane opening-Center
GCA: Ganglion cell analysis
GCL: Ganglion cell layer
GMPE: Glaucoma Module Premium Edition
ILM: Internal limiting membrane
IOP: Intraocular pressure
IPL: Inner plexiform layer
OCT: Optical coherence tomography
ONH: Optic nerve head
OR: Odds ratio
RNFL: Retinal nerve fiber layer
VF: Visual field

## References

[1] Huang D (1991). Optical coherence tomography. Science.

[2] Hee MR (1995). Optical coherence tomography of the human retina. Arch. Ophthalmol..

[3] Swanson EA (1993). In vivo retinal imaging by optical coherence tomography. Opt. Lett..

[4] Schuman JS (1995). Optical coherence tomography: A new tool for glaucoma diagnosis. Curr. Opin. Ophthalmol..

[5] Lederer DE (2003). Analysis of macular volume in normal and glaucomatous eyes using optical coherence tomography. Am. J. Ophthalmol..

[6] Guedes V (2003). Optical coherence tomography measurement of macular and nerve fiber layer thickness in normal and glaucomatous human eyes. Ophthalmology.

[7] Wollstein G (2004). Optical coherence tomography (OCT) macular and peripapillary retinal nerve fiber layer measurements and automated visual fields. Am. J. Ophthalmol..

[8] Bussel II, Wollstein G, Schuman JS (2014). OCT for glaucoma diagnosis, screening and detection of glaucoma progression. Br. J. Ophthalmol..

[9] Wollstein G (2005). Optical coherence tomography longitudinal evaluation of retinal nerve fiber layer thickness in glaucoma. Arch. Ophthalmol..

[10] Leung CK (2010). Evaluation of retinal nerve fiber layer progression in glaucoma: a study on optical coherence tomography guided progression analysis. Invest. Ophthalmol. Vis. Sci..

[11] Na JH (2012). Detection of glaucoma progression by assessment of segmented macular thickness data obtained using spectral domain optical coherence tomography. Invest. Ophthalmol. Vis. Sci..

[12] Varma R, Lee PP, Goldberg I, Kotak S (2011). An assessment of the health and economic burdens of glaucoma. Am. J. Ophthalmol..

[13] Tham YC (2014). Global prevalence of glaucoma and projections of glaucoma burden through 2040: A systematic review and meta-analysis. Ophthalmology.

[14] Wang W, He M, Li Z, Huang W (2019). Epidemiological variations and trends in health burden of glaucoma worldwide. Acta Ophthalmol..

[15] Blindness GBD, Vision Impairment Collaborators, Vision Loss Expert Group of the Global Burden of Disease Study (2021). Causes of blindness and vision impairment in 2020 and trends over 30 years, and prevalence of avoidable blindness in relation to VISION 2020: The Right to Sight: An analysis for the Global Burden of Disease Study. Lancet Glob. Health.

[16] Kim KE, Jeoung JW, Park KH, Kim DM, Kim SH (2015). Diagnostic classification of macular ganglion cell and retinal nerve fiber layer analysis: Differentiation of false-positives from glaucoma. Ophthalmology.

[17] Hwang YH, Jeong YC, Kim HK, Sohn YH (2014). Macular ganglion cell analysis for early detection of glaucoma. Ophthalmology.

[18] Kim NR (2011). Factors associated with false positives in retinal nerve fiber layer color codes from spectral-domain optical coherence tomography. Ophthalmology.

[19] Leal-Fonseca M, Rebolleda G, Oblanca N, Moreno-Montanes J, Munoz-Negrete FJ (2014). A comparison of false positives in retinal nerve fiber layer, optic nerve head and macular ganglion cell-inner plexiform layer from two spectral-domain optical coherence tomography devices. Graefes Arch. Clin. Exp. Ophthalmol..

[20] Aksoy FE (2020). A comparative evaluation of segmental analysis of macular layers in patients with early glaucoma, ocular hypertension, and healthy eyes. J. Fr. Ophtalmol..

[21] Kim EK, Park HL, Park CK (2017). Segmented inner plexiform layer thickness as a potential biomarker to evaluate open-angle glaucoma: Dendritic degeneration of retinal ganglion cell. PLoS ONE.

