# Supplementary material for: False-positive classification and associated factors in segmented macular layers and retinal nerve fiber layer analysis: Spectralis OCT deviation map study
Source: Sci Rep. 2023 Apr 25;13:6782. doi: 10.1038/s41598-023-33944-z (PMC10130102; doi:10.1038/s41598-023-33944-z)
Supplement: Supplementary file 1 — Supplementary Information. [file 41598_2023_33944_MOESM1_ESM.pdf]

Supplementary Table 1. Comparison of demographics and characteristics among different false-positive-pattern groups on ganglion cell layer deviation map

	**Group A (n = 43)**	**Group B (n = 9)**	**Group C (n = 5)**	**P value**	**Post hoc test[c]**
Age, y	49.3 ± 16.7 (20–80)	63.2 ± 13.1 (37–80)	75.6 ± 5.7 (66–80)	**0.001**[a]	A<B=C
Male, n (%)	25 (58.1)	4 (44.4)	3 (60.0)	0.817[b]	
Right eye, n (%)	25 (58.1)	6 (66.7)	3 (60.0)	0.900[b]	
IOP, mmHg	14.1 ± 3.1 (8–23)	14.3 ± 2.1 (11–18)	13.8 ± 2.2 (11–16)	0.884[a]	
CCT, μm	549.9 ± 38.5 (465–604)	537.7 ± 27.6 (493–573)	552.6 ± 41.2 (515–597)	0.565[a]	
AXL, mm	25.1 ± 1.8 (22.3–29.4)	24.5 ± 1.9 (21.9–26.9)	22.5 ± 0.0 (22.5–22.5)	0.078[a]	
Refractive error, D	-3.0 ± 3.8 (-11.8–3.3)	-1.8 ± 3.6 (-8.5–2.0)	0.5 ± 0.6 (-0.1–1.0)	0.179[a]	
FoBMOC axis, º	-6.2 ± 3.6 (-15.1–1.5)	-5.1 ± 3.9 (-10.3–3.6)	-5.4 ± 1.5 (-7.4–-3.4)	0.838[a]	
BMO area, mm^2	2.28 ± 0.55 (1.16–3.53)	2.48 ± 0.73 (1.95–4.13)	2.34 ± 0.19 (2.01–2.50)	0.671[a]	
BMO-MRW, μm	253.0 ± 43.8 (131–341)	228.0 ± 29.5 (166–266)	240.8 ± 32.9 (201–273)	0.158[a]	
RNFL thickness, μm	99.6 ± 8.4 (78–115)	97.4 ± 15.9 (75–120)	104.2 ± 6.3 (99–115)	0.493[a]	

AXL axial length, *BMO* Bruch's membrane opening, *CCT* central corneal thickness, *FoBMOC* Fovea-to-BMO-Center, *IOP* intraocular pressure, *MRW* minimum rim width, *RNFL* retinal nerve fiber layer.

Data are mean ± standard deviation unless otherwise indicated.

Boldface indicates $p < 0.05$.

False–positive patterns were classified into 3 groups according to the shape and area of the abnormal color–coded area: group A (island shape circumpassing less than 180° in area); group B (hook shape circumpassing more than 180° but less than 360° in area); group C (donut shape around inner annulus, circumpassing 360° in area).

[a]Kruskal–Wallis test.

[b]Fisher's exact test.

[c]Mann–Whitney U post hoc test.

Supplementary Table 2. Comparison of demographics and characteristics among different false-positive-pattern groups on inner plexiform layer deviation map

	**Group A (n = 39)**	**Group B (n = 5)**	**Group C (n = 2)**	**P value**	**Post hoc test[c]**
Age, y	51.9 ± 17.6 (20–80)	71.6 ± 5.2 (66–76)	72.0 ± 11.3 (64–80)	**0.013[a]**	A<B, A=C, B=C
Male, n (%)	17 (43.6)	2 (40.0)	2 (100.0)	0.385[b]	
Right eye, n (%)	22 (56.4)	3 (60.0)	2 (100.0)	0.818[b]	
IOP, mmHg	13.6 ± 2.7 (8–21)	14.8 ± 1.1 (13–16)	11.0 ± 0.0 (11–11)	0.092[a]	
CCT, μm	545.3 ± 36.4 (465–604)	546.4 ± 46.2 (503–597)	537.5 ± 2.1 (536–539)	0.902[a]	
AXL, mm	25.1 ± 1.9 (22.3–29.4)	22.3 ± 0.3 (21.9–22.5)	25.3 ± 0.0 (25.3–25.3)	**0.024[a]**	A>B, A=C, B=C
Refractive error, D	-3.0 ± 4.0 (-11.8–3.3)	1.0 ± 0.8 (0.5–2.0)	-1.5 ± 1.9 (-2.9–-0.1)	0.112[a]	
FoBMOC axis, º	-6.6 ± 3.8 (-15.1–2.4)	-3.9 ± 4.5 (-7.4–3.6)	-3.9 ± 0.8 (-4.5–-3.3)	0.226[a]	
BMO area, mm^2	2.34 ± 0.56 (1.16–4.13)	2.26 ± 0.26 (1.95–2.50)	2.21 ± 0.30 (1.99–2.42)	0.924[a]	
BMO-MRW, μm	253.9 ± 44.2 (131–341)	237.6 ± 33.2 (201–273)	230.5 ± 23.3 (214–247)	0.431[a]	
RNFL thickness, μm	101.3 ± 9.7 (78–120)	101.6 ± 11.2 (84–115)	87.0 ± 17.0 (75–99)	0.369[a]	

AXL axial length, *BMO* Bruch's membrane opening, *CCT* central corneal thickness, *FoBMOC* Fovea-to-BMO-Center, *IOP* intraocular pressure, *MRW* minimum rim width, *RNFL* retinal nerve fiber layer.

Data are mean ± standard deviation unless otherwise indicated.

Boldface indicates $p < 0.05$.

False–positive patterns were classified into 3 groups according to the shape and area of the abnormal color–coded area: group A (island shape circumpassing less than 180° in area); group B (hook shape circumpassing more than 180° but less than 360° in area); group C (donut shape around inner annulus, circumpassing 360° in area).

[a]Kruskal–Wallis test.

[b]Fisher's exact test.

[c]Mann–Whitney U post hoc test.

Supplementary Table 3. Comparison of demographics and characteristics among different false-positive-pattern groups on retinal layer deviation map

	**Group A (n = 12)**	**Group B (n = 9)**	**Group C (n = 7)**	**P value**
Age, y	55.2 ± 20.2 (21–80)	62.4 ± 17.3 (30–80)	53.4 ± 14.0 (30–66)	0.489[a]
Male, n (%)	6 (50.0)	5 (55.6)	1 (14.3)	0.220[b]
Right eye, n (%)	5 (41.7)	6 (66.7)	4 (57.1)	0.567[b]
IOP, mmHg	14.9 ± 3.4 (10–23)	13.1 ± 2.3 (11–17)	14.1 ± 1.3 (12–16)	0.340[a]
CCT, μm	540.7 ± 42.2 (465–596)	544.6 ± 37.2 (503–597)	528.0 ± 27.5 (493–579)	0.666[a]
AXL, mm	25.5 ± 2.3 (22.5–29.4)	24.6 ± 1.9 (21.9–27.2)	24.6 ± 1.8 (22.5–26.8)	0.616[a]
Refractive error, D	-4.4 ± 5.0 (-11.8–2.9)	-2.8 ± 3.9 (-9.9–2.0)	-2.9 ± 4.2 (-10.1–0.6)	0.722[a]
FoBMOC axis, º	-7.2 ± 4.1 (-14.7–0.0)	-3.9 ± 3.9 (-8.1–3.6)	-5.3 ± 3.2 (-10.3–-0.9)	0.283[a]
BMO area, mm^2	2.46 ± 0.80 (1.48–4.13)	2.02 ± 0.31 (1.37–2.42)	2.35 ± 0.46 (1.66–3.17)	0.307[a]
BMO-MRW, μm	257.6 ± 25.2 (220–315)	255.1 ± 32.1 (214–314)	224.0 ± 45.3 (166–293)	0.167[a]
RNFL thickness, μm	105.3 ± 8.4 (93–115)	97.4 ± 13.1 (75–115)	98.7 ± 11.9 (83–120)	0.277[a]

AXL axial length, *BMO* Bruch's membrane opening, *CCT* central corneal thickness, *FoBMOC* Fovea-to-BMO-Center, *IOP* intraocular pressure, *MRW* minimum rim width, *RNFL* retinal nerve fiber layer.

Data are mean ± standard deviation unless otherwise indicated.

Boldface indicates $p < 0.05$.

False–positive patterns were classified into 3 groups according to the shape and area of the abnormal color–coded area: group A (island shape circumpassing less than 180° in area); group B (hook shape circumpassing more than 180° but less than 360° in area); group C (donut shape around inner annulus, circumpassing 360° in area).

[a]Kruskal–Wallis test.

[b]Fisher's exact test.
